# Activation of ATM/Chk1 by curcumin causes cell cycle arrest and apoptosis in human pancreatic cancer cells

**DOI:** 10.1038/sj.bjc.6605039

**Published:** 2009-04-28

**Authors:** R P Sahu, S Batra, S K Srivastava

**Affiliations:** 1 Department of Pharmaceutical Sciences and Cancer Biology Center, School of Pharmacy, Texas Tech University Health Sciences Center, Amarillo, Texas, USA

**Keywords:** curcumin, pancreatic cancer, G2/M arrest, DNA damage, Chk1

## Abstract

Curcumin has been shown to inhibit the growth of various types of cancer cells; however, at concentrations much above the clinically achievable levels in humans. The concentration of curcumin achieved in the plasma after oral administration in humans was estimated to be around 1.8 *μ*M. Here, we report that treatment of BxPC-3 human pancreatic cancer cells with a low and single exposure of 2.5 *μ*M curcumin for 24 h causes significant arrest of cells in the G2/M phase and induces significant apoptosis. Immunoblot studies revealed increased phosphorylation of H2A.X at Ser-139 and Chk1 at Ser-280 and a decrease in DNA polymerase-*β* level in curcumin-treated cells. Phosphorylation of H2A.X and Chk1 proteins are an indicator of DNA damage whereas DNA polymerase-*β* plays a role in the repair of DNA strand breaks. Normal immortalised human pancreatic ductal epithelial (HPDE-6) cells remained unaffected by curcumin treatment. In addition, we also observed a significant increase in the phosphorylation of Chk1 at Ser-345, Cdc25C at Ser-216 and a subtle increase in ATM phosphorylation at Ser-1981. Concomitant decrease in the expressions of cyclin B1 and Cdk1 were seen in curcumin-treated cells. Further, curcumin treatment caused significant cleavage of caspase-3 and PARP in BxPC-3 but not in HPDE-6 cells. Silencing ATM/Chk1 expression by transfecting BxPC-3 cells with ATM or Chk1-specific SiRNA blocked the phosphorylation of ATM, Chk1 and Cdc25C and protected the cells from curcumin-mediated G2/M arrest and apoptosis. This study reflects the critical role of ATM/Chk1 in curcumin-mediated G2/M cell cycle arrest and apoptosis in pancreatic cancer cells.

Pancreatic cancer is the fourth leading cause of cancer-related deaths in the United States (Jemal *et al*, 2005). The poor prognosis in pancreatic cancer is due to the reduced response of patients to chemotherapy and/or radiation therapy. Curcumin (diferuloylmethane) is a major constituent of turmeric powder, which is extracted from the rhizomes of the plant curcuma longa. Many pharmacological and clinical studies support the fact that curcumin has chemopreventive and antiproliferative activity against a variety of human cancers including pancreatic cancers (Ammon and Wahl, 1991; Li *et al*, 2004; Lev-Ari *et al*, 2006; Mitra *et al*, 2006; Reddy *et al*, 2006; Wang *et al*, 2006; Aggarwal *et al*, 2007; Bachmeier *et al*, 2007; Hauser *et al*, 2007; Shankar and Srivastava, 2007; Wahl *et al*, 2007). In addition, curcumin is also pharmacologically safe as it is a naturally occurring compound used as a food-colouring agent and in traditional medicines to treat various diseases in Asian countries (Ammon and Wahl, 1991; Goel *et al*, 2008). Epidemiological studies also support the notion that populations from East Asian countries where the consumption of curcumin is high are at a reduced risk to various types of cancers including pancreatic cancer (Ammon and Wahl, 1991; Goel *et al*, 2008) as compared with the populations of Western countries (Mukhopadhyay *et al*, 2001; Sinha *et al*, 2003). Previous reports have documented that curcumin has anti-inflammatory, antimicrobial, antioxidative, immunomodulating and antiatherogenic properties (Mukhopadhyay *et al*, 2001; Miquel *et al*, 2002; Banerjee *et al*, 2003). Inhibition of cell growth and induction of apoptosis is the common mechanism by which curcumin shows its anticancer effects. Accumulating evidence suggests the involvement of multiple-signaling pathways by which curcumin causes growth suppression of human cancer cells (Cheng *et al*, 2001; Hidaka *et al*, 2002; Bharti *et al*, 2003; Kim *et al*, 2003; Shishodia *et al*, 2003; Blasius *et al*, 2006; Lev-Ari *et al*, 2006; Mitra *et al*, 2006; Park *et al*, 2006; Tan *et al*, 2006; Aggarwal *et al*, 2007; Aoki *et al*, 2007; Deeb *et al*, 2007; Fahey *et al*, 2007; Lin *et al*, 2007, 2008; Marín *et al*, 2007; Shankar and Srivastava, 2007; Srivastava *et al*, 2007; Weir *et al*, 2007; Binion *et al*, 2008; Freudlsperger *et al*, 2008; Ji *et al*, 2008; Kasinski *et al*, 2008; Mackenzie *et al*, 2008; Shankar *et al*, 2008; Sun *et al*, 2008). Phase I clinical trials of curcumin demonstrated encouraging chemopreventive effects in patients with high-risk or pre-malignant lesions. It is also non-toxic to humans up to the dose of 8 g day^−1^ (Cheng *et al*, 2001; Sharma *et al*, 2004).

Although the anticancer effects of curcumin have been documented in various types of cancers, no evidence is available on curcumin-mediated cell cycle regulation in pancreatic cancer. We show herein a novel mechanism by which curcumin causes G2/M cell cycle arrest and apoptosis in pancreatic cancer cells at concentrations that are very close to plasma-achievable concentrations of curcumin in humans. Our studies also identify Chk1 as a novel molecular target of curcumin in pancreatic cancer cells.

## Materials and methods

### Chemicals

Curcumin, RNase A, propidium iodide and antibody against *β* actin were purchased from Sigma-Aldrich (St Louis, MO, USA). Heat inactivated fetal bovine serum and RPMI-1640 medium was obtained from Mediatech Cell Grow (Herndon, VA, USA). Electrophoresis reagents were from Amresco (Solon, OH, USA). Antibodies against phospho–ataxia–telangiectasia-mutated (ATM) (Ser-1981), ataxia–telangiectasia-mutated Rad3 related (ATR) (Ser-428), check point kinase-1 (Chk1) (Ser-345, 317, 296 and 280), check point kinase-2 (Chk2) (Thr-68), cell division cycle (Cdc25C) (Ser-216), H2A.X (Ser-139) as well as against total protein of ATM, ATR, Chk1, Chk2, Cdc25C, Cdk1, CyclinB1, cleaved fragments of caspase-3, poly(ADP-ribose) polymerase (PARP) and human-specific Signal Silence Chk1-SiRNA kit were procured from Cell Signaling Technology Inc. (Danvers MA, USA). Human-specific ATM-SiRNA was procured from Santa Cruz Biotechnology Inc. (Santa Cruz, CA, USA). Transfection reagent (TransIT-TKO) was from Mirus Bio Corporation (Madison, WI, USA), whereas, antibody against DNA polymerase *β*-1 was obtained from Lab Vision Corporation (Fremont, CA, USA). Chemicals for cell culture such as penicillin/streptomycin antibiotic mixture (PSN), sodium pyruvate, HEPES buffer, opti-mem I-reduced serum medium were purchased from GIBCO BRL (Carlsbad, CA, USA). Cell death detection apoptosis ELISA kit was the product of Roche Applied Science (Mannheim, Germany), whereas enhanced chemiluminiscence reagent for western blotting was purchased from Perkin Elmer (Waltham, MA, USA).

### Cell culture and proliferation assays

BxPC-3 cells (a well-differentiated epithelial pancreatic adenocarcinoma cell line obtained from a male Caucasian donor having mutant p53 and wild-type K-ras) were obtained from American Type Cell Culture. A monolayer culture of BxPC3 cells was maintained in RPMI-1640 medium supplemented with 10% fetal bovine serum, 4.5% glucose, 10% sodium pyruvate, 10% HEPES and antibiotics in a humidified incubator with 5% CO_2_ and 95% air. Normal human pancreatic ductal epithelial cells (HPDE-6) were a generous gift from Dr Ming-Sound Tsao (Toronto, Canada). The long-term culture of pancreatic ductal epithelial cells derived from normal and benign adult human pancreata was achieved by infection with a retrovirus containing the E6 and E7 genes of the human papilloma virus 16 (Furukawa *et al*, 1996; Ouyang *et al*, 2000). HPDE-6 cells were maintained in keratinocyte-SFM serum-free medium supplemented with 4 mM L-glutamine and adjusted to contain 0.2 ng ml^−1^ EGF, 30 *μ*g ml^−1^ BPE and 1% (v v^−1^) PSN (Furukawa *et al*, 1996; Ouyang *et al*, 2000). A stock solution of curcumin was prepared in dimethyl sulfoxide (DMSO), which was subsequently diluted in medium so that the concentration of DMSO was less than 0.1%. BxPC-3 and HPDE-6 cells were treated with varying concentrations of curcumin for 24 h. The effect of curcumin on survival of BxPC3 cells was determined by Sulforhodamine B assay as described earlier (Zhang *et al*, 2006; Sahu and Srivastava, 2009). The plates were read at 570 nm with a Bio Kinetics plate reader EL-800 from BioTek Instrument Inc., (Winooski, VA, USA).

### Cell cycle analysis

The effect of curcumin on cell cycle distribution was assessed by flow cytometry after staining the cells with propidium iodide. Briefly, 0.3 × 10^6^ cells (BxPC-3 and HPDE-6) were plated and allowed to attach overnight. The medium was replaced with fresh complete medium containing the differing concentrations of (0, 2.5, 5 and 10 *μ*M) curcumin or DMSO. After incubating for specified times, cells were collected using 0.05% trypsin, washed two times with cold PBS and fixed with ice-cold 70% ethanol overnight at 4°C. The cells were then treated with 80 mg l^−1^ RNase A and 50 mg l^−1^ propidium iodide for 30 min as described earlier (Srivastava and Singh, 2004). The stained cells were analysed using a Coulter Epics XL Flow Cytometer.

### Western blot analysis

BxPC-3 and HPDE-6 cells were treated with varying concentrations of curcumin (0, 2.5, 5 and 10 *μ*M) for 24 h. Whole cell extracts were prepared as described earlier (Zhang *et al*, 2006; Sahu and Srivastava, 2009). Lysates containing 20–40 *μ*g of proteins were subjected to SDS–PAGE followed by transfer of proteins to a PVDF membrane. After blocking with 5% nonfat dry milk for 1 h at RT, membranes were incubated overnight at 4°C with the desired primary antibody (1 : 1000 dilution). Membranes were washed with TBS 0.1% Tween-20 for 20–30 min followed by incubation in a secondary antibody linked to HRP. Immunoreactive bands were visualised using an enhanced chemiluminescence kit according to the manufacturer's instructions. The same membrane was reprobed with the antibody against *β*-actin (1 : 50 000 dilution) as an internal control for equal protein loading.

### Apoptosis determination

Apoptotic cell death was determined by probing with caspase-3 and PARP-cleaved antibodies and by cell death detection ELISA kit as described earlier (Shi *et al*, 2008; Zhang *et al*, 2008). Cell death detection is based on a quantitative sandwich-enzyme immunoassay principle using monoclonal antibodies directed against DNA and histones. Briefly, BxPC-3 cells were seeded in 96-well plates and transfected either with ATM or Chk1-SiRNA followed by treatment with 2.5 *μ*M curcumin for 24 h. The plates were read at 405 nm against sample and at 490 nm for blank on EL800 ELISA plate reader, Bio Tek Instruments. Each sample was analysed in triplicate and the average values were subtracted from the background values.

### Transfection with ATM or Chk1-SiRNA

BxPC-3 cells were transiently transfected either with ATM or Chk1-SiRNA to silence constitutive ATM or Chk1 expression. Briefly 0.3 × 10^6^ cells were transfected either with 50 nM ATM-SiRNA or 100 nM Chk1-SiRNA in opti-mem-reduced serum medium using Mirus TransIT-TKO transfection reagent for 24 h. Following transfection, cells were treated with DMSO or curcumin (2.5 *μ*M) for 24 h. Transfected cells were either processed for cell cycle or western blot analysis.

### Densitometric scanning and statistical analysis

The intensity of immunoreactive bands was determined using a densitometer (Molecular Dynamics, Minneapolis, MN, USA) equipped with Image QuaNT software. Results are expressed as means±s.e.m. of at least two independent experiments, each conducted in triplicate. Data for cell death ELISA and cell cycle were analysed by non-parametric ANOVA followed by Bonferroni's *post hoc* analysis for multiple comparisons. All statistical calculations were performed using InStat software and GraphPad Prizm 4.0. Differences between control and curcumin treatment were analysed by 1-way ANOVA. Differences were considered significant at *P*<0.05.

## Results

### Antiproliferative effect of curcumin

**Figure 1:**
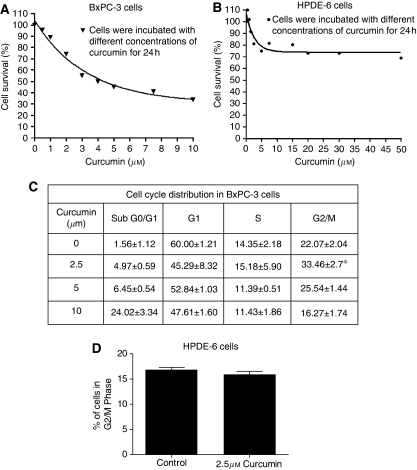
Effect of curcumin on the proliferation and cell cycle distribution of BxPC-3 and HPDE-6 cells. BxPC-3 human pancreatic cancer cells and normal HPDE-6 cells were treated with different concentrations of curcumin for 24h. The effect of curcumin on the proliferation of these cells was analyzed by Sulforhodamine B assay. The values are mean±s.e.m. of 3 independent experiments (each conducted in triplicate) (**A** and **B**). BxPC-3 and HPDE-6 cells were treated with different concentrations of curcumin and its effect on the cell cycle distribution was evaluated by flow cytometry as described in the Materials and Methods (**C** and **D**). The values are means±s.e.m. of 2 independent experiments (each conducted in triplicate). ^*^Different from control, *P*<0.05.

We first determined the optimum dose of curcumin required to inhibit the proliferation of BxPC-3 cells. Treatment of BxPC-3 cells with increasing concentrations of curcumin for 24 h significantly reduced the survival of cells with an IC_50_ of 5±0.5 *μ*M (Figure 1A). On the other hand, survival of normal HPDE-6 cells was minimally affected by curcumin treatment even at concentrations that were highly toxic to BxPC-3 cells.

### Curcumin causes G2/M cell cycle arrest

To gain further insight into the mechanism of the growth inhibitory effects of curcumin, BxPC-3 cells were treated with different concentrations of curcumin and analysed for cell cycle distribution. As shown in Figure 1C, as compared with DMSO-treated cells, 2.5 *μ*M curcumin treatment caused significant arrest of cells in the G2/M phase, whereas higher concentrations of curcumin-induced extensive apoptosis. However, curcumin treatment did not cause any change in the cell cycle distribution of normal HPDE-6 cells (Figure 1D).

### Curcumin causes DNA damage and induces apoptosis

**Figure 2:**
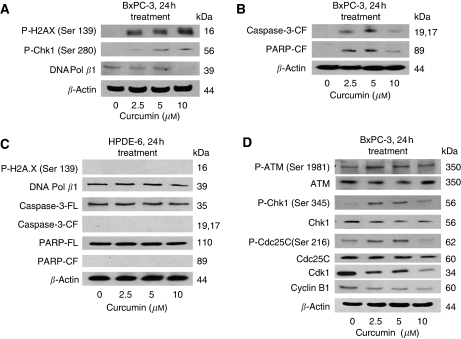
Curcumin treatment causes DNA damage and induces apoptosis in BxPC-3 but not in normal HPDE-6 cells. BxPC-3 cells were treated with different concentrations of curcumin for 24h. Cells were lysed and the total lysate was prepared as described in the Materials and Methods. Representatives immunoblots show the effect of curcumin treatment in BxPC-3 cells on the expression or phosphorylation of H2A.X (Ser-139), Chk1 (Ser-280), DNA polymerase *β* (**A**), cleaved fragments of caspase-3 and PARP (**B**), p-ATM (Ser-1981), p-Chk1 (Ser-345), p-Cdc25C (Ser-216) and protein expression of ATM, Chk1, Cdc25C, Cdk1, Cyclin B1 (**D**). The effect of curcumin was also evaluated in normal HPDE-6 cells on p-H2A.X (Ser-139), DNA polymerase *β*, full length and cleaved fragments of caspase-3 and PARP (**C**). Each blot was stripped and reprobed with anti-*β*-actin antibody to ensure equal protein loading. Intensities of immunoreactive bands were quantified by densitometric scanning.

To determine the DNA-damaging effect of curcumin, cell lysates were analysed by western blotting. Phosphorylation of H2A.X at Ser-139, Chk1 at Ser-280 and Ser-296 are the indicators of the presence of DNA double-strand breaks. We observed an increased phosphorylation of H2A.X at Ser-139 and Chk1 at Ser-280 (Figure 2A), whereas no phosphorylation of Chk1 at Ser-296 was observed in control and treated cells (data not shown). At the same time, our results show that curcumin treatment decreased expression of DNA polymerase *β* (Figure 2A). DNA polymerase *β* plays a crucial role in the repair of DNA strand break. We also observed an increase in the cleaved fragments of caspase-3 and PARP in curcumin-treated BxPC-3 cells, indicating apoptosis (Figure 2C). Nevertheless, we did not observe phosphorylation of H2A.X or Chk1 or cleavage of caspase3/PARP by curcumin treatment in HPDE-6 cells (Figure 2B and D).

### Curcumin treatment modulates expression of G2/M cell cycle regulatory proteins

DNA damage generally leads to the activation of the ATM/ATR pathway (Molinari, 2000; Abraham, 2001; Lukas *et al*, 2001; Huang *et al*, 2008). To further delineate the molecular mechanism of curcumin-mediated G2/M arrest, we determine its effect on the key-signaling proteins of this pathway. Treatment of BxPC-3 cells with 2.5 *μ*M curcumin increased the phosphorylation of ATM at Ser-1981 without any change in the protein level. We did not observe any change in the phosphorylation of ATR at Ser-428 or Chk2 at Thr-68 in curcumin-treated cells (data not shown). On the other hand, substantial phosphorylation of Chk1 at Ser-345 and Cdc25C at Ser-216 was observed in BxPC-3 cells treated with curcumin. For example, 2.5 *μ*M curcumin caused about 6-fold phosphorylation of Chk1 at Ser-345 and 2 fold phosphorylation of Cdc25C at Ser-216 respectively when compared with their respective controls. Protein expression of Chk1 and Cdc25C, however, remained unaltered during the treatment (Figure 2D). The activation of Cdk1/Cyclin B1 complex is the rate-limiting factor for the cells to enter into mitosis, whereas its inactivation leads to G2/M arrest (Jackman *et al*, 2003). Exposure of BxPC-3 cells with 2.5 *μ*M curcumin for 24 h significantly reduced the expression of Cdk1 and Cyclin B1 as compared with DMSO-treated control cells (Figure 2D). These results suggest the possible involvement of ATM/Chk1/Cdc25C and downregulation of Cdk1 and cyclin B1 in curcumin-mediated G2/M cell cycle arrest.

### Silencing of ATM or Chk1 protein expression attenuate G2/M arrest and apoptosis by curcumin

**Figure 3:**
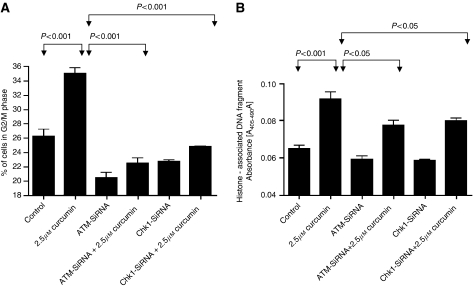
Role of ATM/Chk1 in curcumin mediated G2/M cell cycle arrest and apoptosis. BxPC-3 cells were transiently transfected either with ATM-SiRNA or Chk1-SiRNA followed by treatment with or without 2.5*μ*M curcumin for 24h. Control cells received DMSO only. Cells were collected and analysed for cell cycle distribution by flow cytometry (**A**). In a separate experiment, cells were plated in 96 well plates and transiently transfected either with ATM or Chk1-SiRNA followed by treatment with or without 2.5*μ*M curcumin for 24h. Cells were lysed and analysed for apoptosis by the cell death detection ELISA method according to the manufacturer's protocol (**B**). The values are means±s.e.m. of 2 independent experiments (each conducted in triplicate). Data were analyzed by non-parametric ANOVA followed by Bonferroni's *post hoc* analysis for multiple comparisons. Differences between tested groups were analysed and considered significant at *P*<0.05 from control.

Next we raised a question whether activation of ATM or Chk1 by activating phosphorylation plays any direct role in curcumin-mediated cell cycle arrest and apoptosis. To address this question, we transiently transfected BxPC-3 cells with either ATM or Chk1-SiRNA and then subjected the cells to curcumin treatment for 24 h. Cells were then analysed for cell cycle distribution by flow cytometry and apoptosis by cell death detection ELISA assay. Our results demonstrate that silencing ATM or Chk1 expression completely prevents G2/M cell cycle arrest and protects the cells from curcumin-induced apoptosis (Figure 3A and B). However, it was rather surprising that transfection of cells with ATM-SiRNA alone significantly reduced the percentage of cells in G2/M phase. The explanation for this paradox is not clear at this point and warrants further investigation.

**Figure 4:**
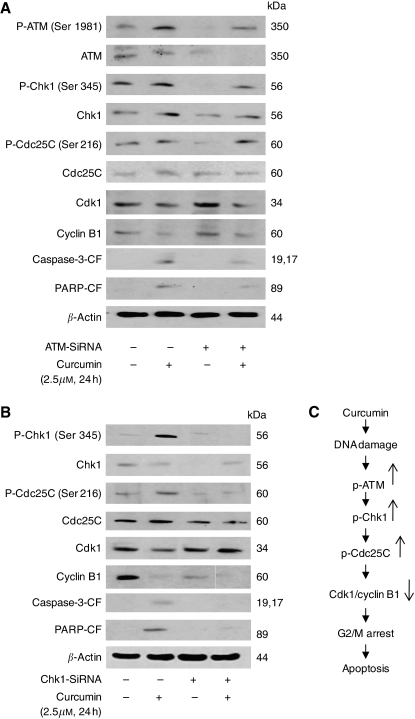
Involvement of ATM/Chk1 in curcumin induced G2/M arrest and apoptosis. BxPC-3 cells were transiently transfected either with ATM-SiRNA (**A**) or Chk1-SiRNA (**B**) followed by treatment with or without 2.5*μ*M curcumin for 24h. Control cells received DMSO only. Total cell lysates were prepared and samples were separated on 10% SDS-PAGE. Samples were analysed to evaluate the expression of phospho-ATM (Ser-1981), Chk1 (Ser-345), Cdc25C (Ser-216) and protein expression of ATM, Chk1, Cdc25C, Cdk1, Cyclin B1 and cleaved fragments of caspase-3 and PARP. Each blot was stripped and reprobed with anti-*β*-actin antibody to ensure equal protein loading. Intensities of immunoreactive bands were quantified by densitometric scanning. Possible mechanism by which curcumin induces G2/M arrest and apoptosis in BxPC-3 cells (**C**).

To further see whether blocking ATM or Chk1 activation can prevent the modulation of G2/M regulatory proteins by curcumin, cells were transfected with ATM or Chk1-SiRNA followed by treatment with 2.5 *μ*M curcumin for 24 h. Our results show that silencing ATM significantly blocked curcumin-mediated activation of ATM at Ser-1981, Chk1 at Ser 345, Cdc25C at Ser 216 and modestly blocked the downregulation of the expression of Cdk1 and Cyclin B1 (Figure 4A). A substantial attenuation was observed in the cleavage of caspase-3 and PARP in ATM-silenced curcumin-treated cells as compared with curcumin treatment alone (Figure 4A).

Similar to ATM, silencing Chk1 by Chk1-specific SiRNA, curcumin-mediated phosphorylation of Chk1 at Ser-345 and Cdc25C at Ser-216 was completely prevented (Figure 4B). The protein level of Cdc25C remained unaltered whereas curcumin-mediated downregulation of Cdk1 and cyclin B1 expression was substantially blocked and was equivalent to the control level (Figure 4B). In addition, cleavage of caspase-3 and PARP by curcumin was also significantly blocked in Chk1-silenced cells (Figure 4B). Taken together our results suggest the involvement of ATM/Chk1 in curcumin-mediated G2/M arrest and apoptosis.

## Discussion

For maintenance of a normal cell cycle, cells possess cell cycle check points as control mechanisms to ensure proper execution of cell cycle events by protecting dividing cells from potentially fatal consequences of DNA damage (Singh and Khar, 2006; Tse *et al*, 2007). During DNA damage, cells are blocked in G2/M phase to provide time to repair damaged DNA (Molinari, 2000; Abraham, 2001), or lead to apoptotic cell death in case of severe DNA damage (Huang *et al*, 2008).

Several studies have indicated that curcumin induces cell cycle arrest and apoptosis in various human cancer cells (Park *et al*, 2006; Tan *et al*, 2006; Srivastava *et al*, 2007; Weir *et al*, 2007; Lin *et al*, 2008; Mackenzie *et al*, 2008; Sun *et al*, 2008). This study investigates the mechanism of DNA damage-mediated cell cycle arrest by curcumin in pancreatic cancer cells. We observed an increase in the phosphorylation of H2A.X at Ser-139, Chk1 at Ser-280 and a downregulation of the DNA polymerase-*β* enzyme, indicating the presence of DNA double-strand breaks. On the other hand, normal HPDE-6 cells did not show any DNA damage incurred by curcumin treatment.

We demonstrate that curcumin treatment causes G2/M cell cycle arrest at a low concentration of 2.5 *μ*M; whereas higher concentrations (5–10 *μ*M) of curcumin-induced extensive apoptosis in BxPC-3 cells as detected by cleavage of caspase-3 and PARP. However, normal HPDE-6 cells were minimally affected by curcumin treatment even at concentrations that were highly toxic to BxPC-3 pancreatic cancer cells. The proximal transducer kinases ATM and ATR both possess the functional properties of a sensor. ATM is phosphoinositide 3-kinase-related kinases that play an important role in cell proliferation and DNA repair (Huang *et al*, 2008). DNA damage check points are predominantly associated with the activation of ATM whereas ATR is activated by stalling of the replication fork induced by UV, nucleotide imbalance, and DNA cross-linking (Lukas *et al*, 2001; Chen *et al*, 2008). During this process, ATM undergoes autophosphorylation on Ser-1981 and is recruited at the sites of DNA damage where it initiates a series of signaling cascades through the phosphorylation of multiple DNA damage response cell cycle proteins including Chk1 at Ser 345 and Ser 317/Chk2 at Thr-68 (Molinari, 2000; Abraham, 2001; Lukas *et al*, 2001). Our results clearly demonstrate the activation of ATM by phosphorylation at Ser-1981 by curcumin treatment. We, however, did not observe the activation of ATR by curcumin. These data are in line with the previous published study where kotomolide, a butanolide constituent isolated from the leaves of C. kotoense induced cell cycle arrest and apoptosis through the activation of ATM in non-small cell lung cancer A549 cells (Huang *et al*, 2008). To show the involvement of ATM in curcumin-mediated cell cycle arrest, we selectively silenced the expression of ATM protein by ATM-specific SiRNAs. Silencing ATM expression significantly abrogated the activation of ATM at Ser-1981, Chk1 at Ser-345 and Cdc25C at Ser-216 in curcumin-treated cells and substantially prevented the cells from undergoing G2/M arrest and apoptosis.

Chk1, upon activation, phosphorylates Cdc25C at Ser-216 leading to inactivation of Cdk1–Cyclin B1 complex, which in turn leads to G2/M arrest (Molinari, 2000; Abraham, 2001; Lukas *et al*, 2001). Two check point kinases, Chk1 and Chk2, although being structurally different from each other in serine/threonine kinases, share overlapping functions (Jackman *et al*, 2003; Herman-Antosiewicz and Singh, 2005; Sidi *et al*, 2008; Wang *et al*, 2008a, 2008b). Over the past many years, an enormous effort has been made to gain insights into cell cycle checkpoint functions. Chk1 is an established transducer of ATR and ATM-dependent signaling in response to DNA damage. In addition to nuclear localisation, its presence on interphase centrosomes negatively regulates entry into mitosis by preventing premature activation of cyclin B-cdk1 complex during unperturbed cell cycles (Tyagi *et al*, 2005). In human cancers, dysfunction in this checkpoint is considered a serious pathologic hallmark of neoplastic transformation (Chen *et al*, 2008). Various chemotherapeutic agents and ionising radiation, which are used to treat cancer, have been shown to activate Chk1 (Chen *et al*, 2008).

We observed significant activation of Chk1 by phosphorylation at Ser-345 with curcumin treatment. These results are in agreement with previous studies where diallyl trisulfide, resveratrol and lithium caused G2/M cell cycle arrest by activation of Chk1 in human prostate (Herman-Antosiewicz and Singh, 2005), ovarian (Tyagi *et al*, 2005) and hepatocellular (Wang *et al*, 2008a, 2008b) carcinoma cells. However, no change in the activation of Chk1 at Ser-317 and Chk2 at Thr-68 was demonstrated by curcumin treatment. We further observed the phosphorylation of Cdc25C at Ser-216 and reduced expression of Cdk1/Cyclin B1 by curcumin treatment as shown earlier in other experimental models (Tyagi *et al*, 2005; Chen *et al*, 2008). To further strengthen the role of Chk1 in G2/M regulation, Chk1 protein expression was specifically silenced in BxPC-3 cells by Chk1-SiRNAs. Silencing Chk1 expression significantly abrogated the activation of Chk1 at Ser-345, Cdc25C at Ser-216 and expression of Cdk1 and Cyclin B1 in curcumin-treated cells and substantially prevented the cells from undergoing G2/M arrest and apoptosis, suggesting that Chk1 plays an important role in curcumin-induced cell cycle arrest and apoptosis. Although our results are consistent with several published reports (Herman-Antosiewicz and Singh, 2005; Tyagi *et al*, 2005; Chen *et al*, 2008), a recent study made slightly conflicting observations. This study observed that abrogation of curcumin-mediated activation of Chk1 and G2/M cell cycle arrest induced apoptosis in hepatoma cells (Wang *et al*, 2008a, 2008b). The reason for this discrepancy could be attributed to the cell-specific effect of curcumin. We found that the curcumin-mediated effect was specific to pancreatic cancer cells as the normal HPDE-6 cells show no change following curcumin treatment. On the basis of these findings, a possible mechanism by which curcumin induces G2/M arrest and apoptosis in BxPC-3 cells is summarised in Figure 4C.

Curcumin has been used in Asian countries as a dietary spice, a food-colouring agent and for the treatment of variety of ailments, including biliary disorder, anorexia, cough, diabetic wounds, hepatic disorders, rheumatism and sinusitis (Furukawa *et al*, 1996; Sharma *et al*, 2004). Although many *in vitro* studies have demonstrated the potential chemotherapeutic effects of curcumin against a variety of cancer cells, its clinical implementation has been a challenge because of its short half life and low bioavailability after oral administration (Cheng *et al*, 2001; Ireson *et al*, 2001; Singh and Khar, 2006). Cheng *et al* (2001) observed an average peak plasma concentration of 0.51–1.77 *μ*M (188–652 ng ml^−1^) after an oral administration of 4–8 g curcumin per day in patients with pre-malignant lesions. It is important to point out that the inhibitory concentration of 2.5 *μ*M curcumin in our model is very close to the clinically achievable plasma concentration of curcumin in humans suggesting its potential for the management of pancreatic cancer.

In addition to the effectiveness of curcumin alone, it is being currently evaluated in combination therapy (Koo *et al*, 2004; Kamat *et al*, 2007; Kunnumakkara *et al*, 2007; Lev-Ari *et al*, 2007; Patel *et al*, 2008). Kunnumakkara *et al* (2007) demonstrated that curcumin can potentiate the antitumour effects of gemcitabine by suppressing the proliferation and angiogenesis in an orthotopic model of pancreatic cancer. They observed that the ability of curcumin to decrease the expression of NF-kB-regulated gene products VEGF, Cyclin D1, c-Myc, ICAM-1, MMP-9, COX-2, survivin, Bcl-2, IAP1 and Bcl-xl in the tumours was enhanced in the combination treatment (Kunnumakkara *et al*, 2007). On the other hand, Lev-Ari *et al* (2006) showed that the antitumour activity of curcumin was associated with the decreased expression of EGFR, COX-2 and ERK in pancreatic cancer cells . These effects were pronounced in the cells P34-expressing COX-2 as compared with low COX-2-expressing Panc-1 cells (Lev-Ari *et al*, 2006).

Limited studies on curcumin in humans have been documented recently (Sharma *et al*, 2004; Dhillon *et al*, 2008). Sharma *et al* (2004), in a phase I clinical trial explored the pharmacology of curcumin in patients with colorectal cancer and suggested that curcumin could be used as an oral cancer preventive or therapeutic agent . Similarly, in a very recent study, Dhillon *et al* (2008) in a phase II clinical trial in patients with advanced pancreatic cancer concluded that 8 g of curcumin given orally was well tolerated and in spite of its limited absorption showed biological activity in some patients . Our preclinical studies also support these clinical studies on curcumin.

To conclude, our present observations state that curcumin treatment potentially inhibits the proliferation of BxPC-3 human pancreatic cancer cells by DNA damage-mediated G2/M cell cycle arrest by the activation of ATM/Chk1/Cdc25C and inhibition of cyclin B1/Cdk1 expression. Our results indicate Chk1 as a novel molecular target of curcumin in pancreatic cancer cells. Nevertheless, further studies are needed to determine the mechanism of DNA damage and pinpoint other pivotal regulators of the signaling pathways mediated by curcumin in human pancreatic cancer cells.
